# Influence of the Model of Care on the Outcomes of Diabetes Self-Management Education Program: A Scoping Review

**DOI:** 10.1155/2021/2969243

**Published:** 2021-02-19

**Authors:** Emmanuel Kumah, Emmanuel K. Afriyie, Aaron A. Abuosi, Samuel E. Ankomah, Adam Fusheini, Godfred Otchere

**Affiliations:** ^1^Policy, Planning, Monitoring, & Evaluation Unit, Komfo Anokye Teaching Hospital, Kumasi, Ghana; ^2^Laboratory Services Directorate, Komfo Anokye Teaching Hospital, Kumasi, Ghana; ^3^Department of Public Administration and Health Services Management, University of Ghana Business School, Ghana; ^4^Department of Preventive and Social Medicine, Dunedin School of Medicine, University of Otago, Dunedin, New Zealand; ^5^Center for Health Literacy and Rural Health Promotion, P.O. Box GP1563, Accra, Ghana

## Abstract

**Background:**

Type 2 diabetes mellitus (T2DM) accounts for approximately 95% of all diabetes cases, making the disease a global public health concern. The increasing prevalence of T2DM has highlighted the importance of evidence-based guidelines for effective prevention, management, and treatment. Diabetes self-management education (DSME) can produce positive effects on patient behaviors and health status. *Study objective*. We synthesized findings from the existing studies to find out whether or not the impact of DSME on patient health behaviors and outcomes differ by the different models of diabetes care. That is, we determined whether there are differences in DSME outcomes when patient's care provider is a general practitioner, a specialist, a nurse, or a combination of these health professionals.

**Methods:**

Searches were made of six electronic databases to identify relevant English language publications on DSME from 2000 through 2019. Titles and abstracts of the search results were screened to select eligible papers for full-text screening. All eligible papers were retrieved, and full-text screening was done by three independent reviewers to select studies for inclusion in the final analysis. Twenty-one studies were included in the final analysis. The main outcome measures assessed were glycated hemoglobin (HbA1c), body mass index (BMI), diet, and physical activity.

**Results:**

The majority of the patients with diabetes were seen by primary care physicians. In general, the studies reported significant improvements in patient health behaviors and outcomes. Some differences in outcomes between the different models of care were observed.

**Conclusion:**

Our findings suggest that the effects of DSME on patients' health behaviors and outcomes could differ by the different models of diabetes care. However, considering the limited sample of publications reviewed, and because none of the reviewed studies directly measured the impact of the DSME program on patient behaviors and outcomes, significant conclusions could not be reached.

## 1. Introduction

Diabetes mellitus (DM) is one of the most common chronic disorders in the world [1]. It is the fifth leading cause of mortality in most high-income countries and rapidly becoming a major health concern in low- and middle-income countries [2]. The global diabetes prevalence in 2019 was estimated at 9.3% (463 million people), and this is estimated to rise to 10.2% (578 million) by 2030 and 10.9% (700 million) by 2045 [3]. There are three main types of diabetes: type 1 diabetes (caused by the body's failure to produce insulin), type 2 diabetes (resulting from insulin resistance), and gestational diabetes (which occurs in pregnant women without previous diagnosis of diabetes) [4]. Type 2 diabetes is the most common type, accounting for approximately 95% of all cases of diabetes [5].

The cost of diabetes care is expensive, and the condition can lead to serious complications such as kidney failure, myocardial infarction, stroke, blindness, and limb amputation [6]. It imposes a huge economic burden on national health care systems globally [1]. That notwithstanding, evidence indicates that early diagnosis and effective management increases the chances of preventing harmful and costly complications associated with diabetes [7]. Evidence has also been established regarding the benefits associated with glycemic control in reducing the risk for and delaying the progression of diabetes complications [8]. Achieving effective glycemic control requires a lifelong adherence to complex lifestyle management, involving regular blood glucose monitoring, self-adjustment of medications, and a physically active lifestyle.

Self-management education (SME) is recognized globally as a tool that helps patients achieve optimum glucose control, through increasing knowledge and awareness, and learning behavioral strategies to manage diabetes [1]. SME is defined as a systematic intervention involving active patient participation in self-monitoring and/or decision-making [9]. Diabetes self-management education (DSME) provides patients with the requisite knowledge and skills to perform self-care behaviors, manage crises, and make lifestyle changes [10]. The program involves different educational, psychological and behavioral interventions; and a combination of didactic, interactive, and collaborative teaching methods tailored to patient's specific needs. Education sessions range from brief instructions by lay leaders, physicians, dieticians, or nurses to more formal and comprehensive programs [7]. Self-efficacy [11], which refers to one's belief in his or her ability to adopt a particular behavior, is a vital component of the concept of SME.

SME is often considered an aspect of patient education. However, the two activities can be distinguished from one another. Patient education focuses on delivering knowledge and skills to patients to enable them to follow medical advice. SME, on the other hand, is concerned with empowering patients to take active control of their illness and apply problem-solving skills to meet new challenges [12].

Many empirical studies [13–18] have demonstrated that DSME programs have a beneficial effect on patients' health status, health behavior, and healthcare utilization, which subsequently reduces the total cost of treating patients with diabetes. In addition, several systematic reviews have shown improvements in outcomes such as better glycemic control, increased weight loss, increased knowledge, decreased blood pressure, improved dietary and exercise habits, and decreased need for diabetes medication [19, 20].

The effectiveness of the DSME program depends on human factors [21, 22], organizational processes [8], and intervention attributes [23, 24]. One other key factor to the success of DSME programs is the influence of patient's routine clinical care provider [8]. Care providers' role—such as serving as conduits for patients to enter the programs, guiding them through the process, and reinforcing what is learned during regular follow-up care—is equally critical to successfully implementing these initiatives [18, 25]. Different health care professionals are responsible for providing care to diabetes patients [26]. However, evidence of whether or not there are differences in DSME outcomes when participant's care provider is a general practitioner, a specialist, a nurse, or a combination of these health professionals has not yet been systematically established.

With this paper, we synthesized findings from the existing literature to determine whether or not the effects of DSME on patients' health behaviors and outcomes differ by the different models of diabetes care. We defined “model of diabetes care” in this study as the type of health professional providing clinical care to patients with diabetes. We focused the review on studies dealing with type 2 DM.

## 2. Models of Routine Diabetes Care

Different models of diabetes care exist in different healthcare settings. One of such models is the specialist service delivery model, involving the use of diabetologists or endocrinologists as providers of diabetes care [27]. The most common model is the primary care physician-led model, where patients with diabetes are managed by primary care physicians [28]. There is also the nurse- and dietitian-led model in which nurses and dietitians, under the supervision of specialists, follow algorithms to deliver education and medical care to patients with diabetes [29]. Other models of diabetes care include advanced nurses and physician-led model [28], nurses and pharmacist-led model [30, 31], clinical pharmacist-led model [32–34], and nurse-led model [35, 36].

Due to the complex nature of diabetes, recent literature emphasizes the application of a team approach to the delivery of care [37–40]. This model of care enables a range of health care providers (primary care physicians, diabetologists, registered nurses, nurse practitioners, physician assistants, certified diabetes educators, dietitians, and pharmacists) to integrate their skills to facilitate improved patient management and outcomes.

## 3. Methods

We used a systematic scoping review, guided by the three-step search strategy recommended by the Joanna Briggs Institute [41] and the PRISMA statement for systematic reviews protocols [42], to gather and summarize the existing literature on the possible influence of the model of diabetes care on the outcomes of DSME interventions. As Arksey and O'Malley have stated that quality assessment does not form part of a scoping review [43], our study does not include assessment of methodological quality of the included papers.

### 3.1. Search Strategy

The search strategy for this review was first drafted for pretesting in (OVID) MEDLINE. Once the MEDLINE strategy was pretested and finalized, it was adapted to the syntax and subject headings of all the other databases searched in the study.  Table 1 demonstrates the search strategy and keywords used (“diabetes mellitus” and “diabetes self-management education”).

The search was conducted between December, 2019 and January, 2020. The following databases and search engines were searched: PubMed, Scopus, MEDLINE, EMBASE, PsychINFO, and the Cochrane Central Register of Controlled Trials. In addition, reference lists of all eligible articles identified were searched and screened for additional relevant studies. We restricted the search to only English language medical literature published between January, 2000 and December, 2019.

### 3.2. Inclusion and Exclusion Criteria

The inclusion criteria are as follows:

- The primary focus of the study should be on self-management education for type 2DM
- The study evaluated the effectiveness of the DSME program on at least one of the following outcome measures: glycated hemoglobin (HbA1c), body mass index (BMI), diet, self-efficacy, mental health, and health service utilization
- The study specified the type of health professional providing routine clinical care to diabetes patients
- The paper was written in the English language

The exclusion criteria are as follows:

- The study is a review article or a report
- The study was not peer-reviewed
- The focus of the paper was on type 1 or both type 1and type 2 diabetes patients
- Participants of the study were type 2 diabetes patients and patients with other chronic conditions such as hypertension and asthma
- The leader of the DSME intervention was at the same time patients' routine clinical care provider

### 3.3. Study Selection Process

Selection and inclusion of papers for this review involved a two-stage process: screening of abstracts and titles and full-text reading to select eligible papers for final inclusion. Three independent reviewers (EK, EKA, and SEA) conducted the selection process through each stage of the review. All publications retrieved through the search were imported into a shared bibliography for duplicate records to be removed. After removing the duplicates, the reviewers applied the predetermined inclusion and exclusion criteria and independently assessed the titles and abstracts for full-text review eligibility. Following this process, articles were selected for full-text screening. Again, the reviewers applied the inclusion and exclusion criteria and independently assessed the full-text articles to select the final set of publications eligible for inclusion in the study. After each stage of the selection process, the reviewers compared results and reached a consensus, with a fourth reviewer (AF) serving as a tiebreaker in an event that the three reviewers failed to reach an agreement.

### 3.4. Data Extraction

Data from the eligible papers were extracted by three members (EK, AAA, and GO) of the research team working independently and checked by a fourth member (AF) to ensure consistency and accuracy of the extracted information. The abstractors documented information on authors and year of publication, sample size, study site (country), study design (randomized controlled trial, quasiexperiment, etc.), intervention type (individual, group, etc.), length of program, program leader (dietitian, nurse, peer educator, physician, etc.), setting of diabetes care (clinic, general medical practice, hospital, etc.), diabetes care provider (general practitioner, specialist, etc.), and study outcomes. We extracted data on the effects of diabetes self-management interventions on glycated hemoglobin (HbA1c), body mass index (BMI), diet, physical activity (aerobic or stretching/strengthening exercise), self-efficacy, mental health (psychological well-being, depression, anxiety, and health distress), and health service utilization (emergency room visits, physician visits, hospital admissions, and length of stay).

## 4. Results

### 4.1. Literature Search

**Figure 1:**
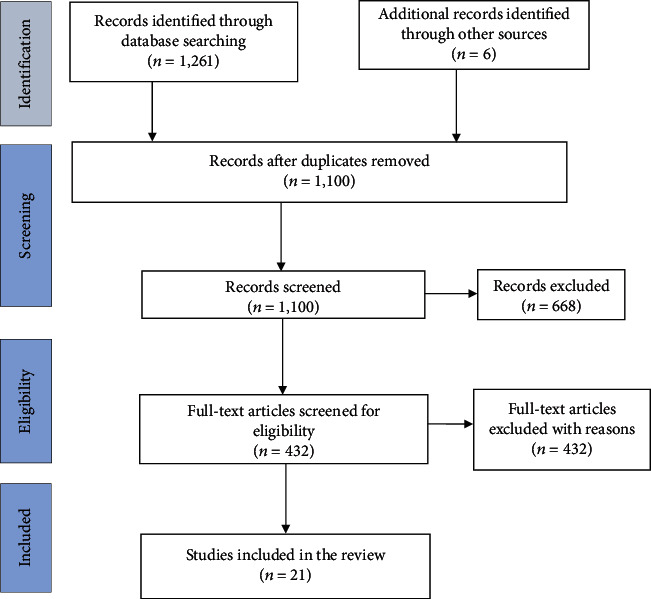
Literature search flow diagram.

The search identified a total of 1,267 papers: 1,261 from the electronic database search and six from the manual search. Following the removal of duplicates, 1,100 articles remained. The abstracts and titles screening resulted in the exclusion of 668 articles, leaving 432 for full-text screening. Four hundred and eleven (411) articles were further excluded after the full-text reading. The most common reason for exclusion was lack of outcome assessment of program effectiveness (*n* = 153). Other common exclusions included article not specifying the name of diabetes care provider (*n* = 41), focusing on either type 1 (*n* = 25), or both type 1 and type 2 (*n* = 97) diabetes, focusing on health professionals and diabetes educators (*n* = 28) and focusing on more than one chronic disease (*n* = 30). In all, 21 articles were included in the final analysis. The flow diagram in Figure 1 depicts stages of study identification and selection.

### 4.2. Description of Studies

Detailed description of the selected studies is presented in Table 2. A total of 4,943 patients with type 2 diabetes were included in the 21 studies. The majority of the studies were conducted in the US (48.9%), randomized controlled trials (57%), group focused (57%), and were professionally led educational programs (76.2%). Most of the studies (15) did not specify the name of the intervention evaluated. The common ones mentioned were the X-PERT and the DESMOND Programs. Detailed information on the interventions evaluated by the included studies is presented in Table 3. Duration of the interventions varied, with the shortest being 6 hours long, delivered between one and two days, and the longest lasting over 2.5 years.

### 4.3. Outcomes

Table 4 displays the setting of care, name of care provider, and the outcomes of interest of the 21 studies. The majority of the interventions (52.4%) were delivered in primary care practice settings, followed by community health facilities (19%) and hospitals (14.3%). Over 60% of the studies (14) included participants receiving care from primary care providers. Three studies mentioned primary care practitioners and nurses as patients' care providers; three indicated specialists as patients' care providers, while one mentioned primary care practitioners and specialists as providers of patients' routine clinical care.

*HbA1c*: twenty studies reported on patients' HbA1c levels; nineteen [44–62] showed statistically significant reductions (-), and one [63] reported no significant improvement (=).

*BMI*: ten studies reported on BMI outcomes; five [44, 46, 58–60] indicated statistically significant positive effects (-), and five [45, 48–50, 61] showed no significant effects (=).

*Diet*: dietary outcomes were reported in five studies; four [48, 56, 57, 62] had positive effects (+), and one [60] indicated no effect (=).

*Physical activity:* this outcome was reported in nine studies; six [46, 48, 57–60] had positive effects (+), and three [56, 62, 63] had no significant effects (=).

*Self-efficacy*: four studies reported on self-efficacy; three [45, 48, 51] indicated positive effects (+), and one [60] showed no significant effect (=).

*Mental health*: mental health was mentioned in four studies; all [56–58, 63] indicating positive outcomes (+).

*Health service utilization*: the only study [64] that reported on health service utilization indicated no significant reduction in health services use (=).

**Figure 2:**
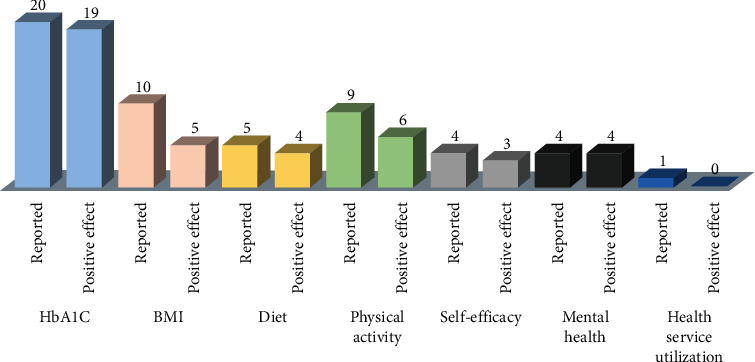
Number of studies reporting on the selected outcome measures and the corresponding number of positive effects on each outcome.

Figure 2 shows the total number of studies that reported on each of the outcome measures, together with the number of positive effects indicated on each outcome.

### 4.4. Model of Care and SME Outcomes

**Figure 3:**
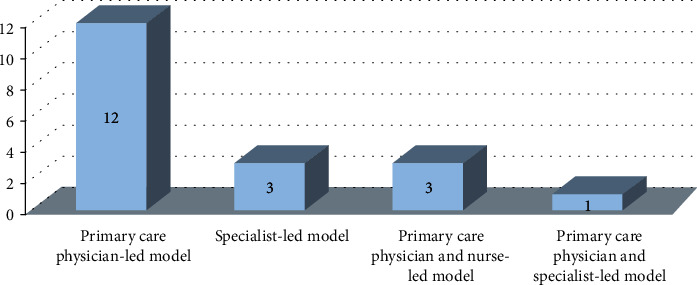
Models of diabetes care.

Based on the setting of care and type of care provider, we identified four models of diabetes care: primary care physician-led model, primary care physician and nurse-led model, primary care physician and specialist-led model, and specialist-led model (Figure 3). Studies that did not mention the specific name of the care provider (e.g., primary care provider) were not included in the models of care classification. Some of the selected studies did not report on all of the outcomes of interest; so, our comparisons were based on four outcome measures: HbA1c, BMI, diet, and physical activity.

**Figure 4:**
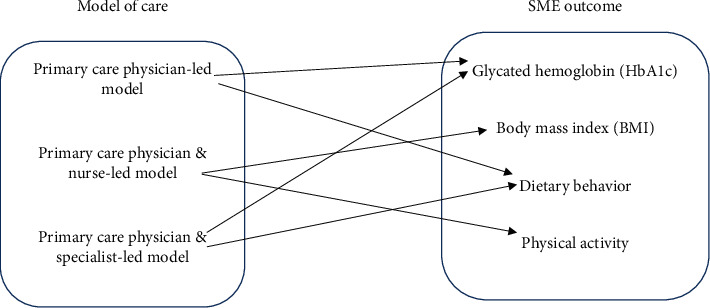
Comparison of positive SME outcomes by the different models of diabetes care. The arrow (→) indicates the positive effect of a model of care on the SME outcome measures. The specialist-led model has been excluded because it had no positive effect on the outcome measures.

Positive effects on HbA1c were reported in both the primary care physician-led model [44, 47–49, 51, 52, 54, 56–59, 64] and the primary care physician and specialist-led model [62] participants' studies, but no statistically significant effects were observed in the specialist-led model [45, 53, 61] and the primary care physician and nurse-led model [46, 55, 60] patients' studies. For instance, in the study by Banister et al. [54] where patients were receiving care under the primary care physician-led model, a significant reduction in mean HbA1c from 9.7 ± −2.4 to 8.2 ± −2.0 was reported. Also, one study under the physician and specialist-led model [62] reported significant reductions in mean HbA1c levels from 8.4 ± 2.3 to 7.6 ± 1.9. Similarly, positive effects on BMI were reported in the primary care physician and nurse-led model participants' studies [46, 55, 60], but no significant effects were shown in both the specialist-led model [45, 53, 61] and the primary care physician-led model [44, 47–49, 51, 52, 54, 56–59, 64] patients' studies. Again, the primary care physician-led model [44, 47–49, 51, 52, 54, 56–59, 64] and the primary care physician and specialist-led model [62] patients' studies reported positive effects on dietary behaviors, while the primary care physician and nurse-led model participants' studies [46, 55, 60] showed no significant improvements in patients' dietary behaviors. Finally, SME interventions where patients were receiving care under the primary care physician and nurse-led model [46, 55, 60] were more effective on physical activity levels than did interventions where participants' care providers were primary care physicians and specialists combined (primary care physician and specialist-led model) (64].  Figure 4 depicts the comparison of the SME outcomes by the models of care.

## 5. Discussion

Diabetes is a complex, chronic condition that requires both high quality clinical care and effective self-management. Different healthcare professionals are responsible for providing clinical care to patients with type 2 diabetes, but the literature is imprecise on whether there are differences in DSME outcomes when the care provider is a GP, a specialist, a nurse, a pharmacist, or a dietitian. We, therefore, synthesized information from the existing literature to ascertain whether DSME programs implemented in patient populations with different care models produce different outcomes.

Generally, the outcomes reported by the studies showed positive effects. Twenty out of the 21 studies reported positive effects on at least one of the outcome measures selected for this study. No study reported that patients' health status deteriorated after participating in the SME programs. Few studies indicated no statistically significant effect on some of the outcome measures. Our findings thus support the literature that DSME programs produce beneficial effects on patients' health behaviors and outcomes [19, 20].

We observed some differences in outcomes between the different models of care. One factor that could explain these differences is the level of participatory decision-making that might have existed between the study participants and their care providers. A participatory relationship between care providers and diabetic patients promotes healthy behaviors [65]. Thus, the studies in which the participants' care providers allowed them to participate fully in treatment decisions might have contributed to their improved health behaviors and outcomes. Even though the selected studies did not provide information on collaboration between patients and their care providers, available evidence in the literature supports our assertion. For instance, Golin et al. [66] found that patients' participation in decision-making increased their self-efficacy levels. Roter [67] noted that self-management improved when the opinions and values of patients were considered in making treatment decisions. Schillinger et al. [26] observed that patients whose care providers asked them to restate the providers' instructions had lower HbA1c levels than patients who were not given the opportunity to restate what they were told. In a study of 752 diabetic patients, effective patient-provider communication was associated with healthier self-reported behaviors such as physical activity, foot care, and dietary adherence [68].

Another factor that might account for these differences could be the degree of collaboration that existed between the care providers and the DSME instructors. SME programs that foster effective collaboration between patients' care providers and self-management instructors report better outcomes [69]. For instance, positive effects were reported on all of the outcome measures (diet, physical activity, HbA1c, and mental health) in one of the selected studies [57], where the authors indicated that patients' care providers received quarterly reports from self-management instructors. A study by Garber et al. [70] also found that effective collaboration between care providers and self-management instructors resulted in overcoming barriers to improving HbA1c levels. Available evidence indicates that the most effective SME programs are those that are well integrated into the health system [71]. This is because SME programs that are integrated into patients' usual care appear to foster better and more effective collaboration between self-management instructors and patients' care providers than do programs that are organized separately from the health system [72]. Thus, the role of health professionals is critical to the success of SME initiatives.

We observed that the majority of the studies (90.5%) included in this review were conducted in high-income countries (HICs). Only two [49, 61] were conducted in low- and middle-income countries (LMICs), and none was conducted in sub-Saharan Africa. This therefore calls for more studies on SME programs in LMICs, especially countries in sub-Saharan Africa.

## 6. Study Limitations

Although only studies published in peer-reviewed journals were considered, the limitations of this review are worth acknowledging. A first limitation relates to the rigorous inclusion and exclusion criteria we adopted. For instance, restricting the search strategy to only English language publications may have resulted in relevant information in studies published in other languages being excluded from our analysis. A second limitation pertains to the limited information on the level of collaboration that existed between patients' care providers and self-management educators. This did not allow us to do a comprehensive analysis of the impact of care providers' involvement in SME interventions on programs' outcomes. Further, the inclusion of studies with different research designs (e.g., randomized controlled trial, quasiexperimental, retrospective case control, and single cohort time-series design) could have implications for the findings synthesized from these studies. The final and the most important limitation relates to the limited sample of publications reviewed. For instance, only three studies each were classified under the specialist-led and the primary care physician and nurse-led models. Also, only one study was found under the primary care physician and specialist-led model. This limits the comparison we made across the different models of diabetes care. Thus, significant conclusions could not be reached. That is, the conclusions drawn are suggestive rather than being conclusive. These limitations notwithstanding, our study provides an important starting point for further investigations into the possible influence of the model of care on the outcomes of DSME programs.

## 7. Conclusions

The differences we observed suggest that the effects of diabetes SME on patients' health behaviors and outcomes could differ by the different models of diabetes care. This therefore underscores the need to take into consideration patients' routine clinical care providers during the design and implementation of DSME interventions. It is also important for researchers, evaluating the effectiveness of SME interventions, to take into account the possible influence of care providers on program effects. However, because none of the studies reviewed directly measured the association between the model of care and the impact of DSME programs on patient behaviors and outcomes, the conclusion drawn should be interpreted with caution. Future studies should consider testing this association. As no standardized and recognized universal patient education considered effective for all individuals has been defined, and countries are finding ways of providing more cost effective SME interventions, findings from this review offer valuable information to healthcare managers, clinicians, and policy makers. The present study adds to and extends the existing knowledge on factors influencing the effectiveness of DSME programs. It also contributes to the optimal design, implementation, and evaluation of effective self-management interventions.

## Figures and Tables

**Table 1:** Detailed search strategy for the scoping review of Diabetes SME interventions.

Search	String
1.	Type 2 diabetes mellitus.mp. or exp noninsulin-dependent diabetes mellitus
2.	Diabetes mellitus.mp. or exp diabetes mellitus/
3.	#1 OR #2
4.	Diabetes self-management.mp.
5.	Diabetes self-management education.mp. or exp diabetes self-management education/or exp patient self-management education/or exp self-care/
6.	Self-management program.mp. or exp self-management intervention/
7.	#4 OR #5 OR #6
8.	#3 AND #7

**Table 2:** Characteristics of selected studies.

Study	Country of study	Sample size	Design	Program name	Type of intervention	Program leader	Program duration
Merakou et al. [44]	Greece	193	CCT	N/A	G	Trained health visitors	4 months
Kazawa et al. [45]	Japan	62	Non-RCT	N/A	I	Nurses	12 months
Dyson et al. [46]	UK	39	RCT	Video education	O	Nurses	6 months
Brunisholz et al. [47]	US	1,920	Retrospective case control	N/A	G and I	Nurses and dietitians	12 months
Kazawa & Moriyama [48]	Japan	30	Pre- and posttest design	N/A	I and O	Nurses	6 months
Gagliardino et al. [49]	Argentina	198	RCT	N/A	G	Peer educators	4 weeks
Rygg et al. [50]	Norway	146	RCT	N/A	G	Nurses	2 to 4 weeks
Yeung et al. [64]	US	60	Single cohort time-series design	Lifelong management	G	Diabetes educator and clinical psychologist	2.5 years
Davies et al. [63]	UK	824	RCT	DESMOND	G	Trained healthcare professional educators	1 day or 2 half days
Pena-Purcell et al. [51]	US	139	Quasiexperimental	N/A	G	Nurses and dieticians	5 weeks
Huang et al. [52]	Korea	154	RCT	N/A	I	Nurses and dietitians	1 year
Song et al. [53]	Korea	31	Quasiexperimental	N/A	G and I	Nurse, dietician, and a physician	10 months
Banister et al. [54]	US	54		N/A	G	Diabetes educators and dietitian	1 year
Goudswaard et al. [55]	Netherlands	54	RCT	N/A	G	Nurses	6 months
Samuel-Hodge et al. [56]	US	117	RCT	A new DAWN	G, I, and O	Peer educators	8-month
Glasgow et al. [57]	US	320	RCT	Diabetes network SME	O	Online professional coach	
Rickheim et al. [58]	US	170	RCT	N/A	G and 1	Educators	6 months
Deakin et al. [59]	UK	157	RCT	X-PERT	G	Dieticians	6 weeks
Vincent [60]	US	20	RCT	N/A	G	Diabetes educators and dietitian	8 weeks
Scain et al. [61]	Brazil	104	RCT	N/A	G	Nurses	4 weeks
Two Feathers [62]	US	151	Non-RCT	N/A	G	Trained family health advocates	4 weeks

CCT: clinically controlled trial; RCT: randomized controlled trial; G: group; I: individual; O: other method, e.g., telephone, mail, online, and video.

**Table 3:** Description of the interventions examined.

Study	Intervention
Merakou et al. [44]	6-hour educational program; two hours per week, and spread in three sessions over a period of 3 weeks
Kazawa et al. [45]	12 months educational program incorporating behavior modification theories such as the transtheoretical model, motivation interviewing, and social support theory
Dyson et al. [46]	Video education—the patients watched three lifestyle videos in their own time
Brunisholz et al. [47]	12 months educational program involving instructions in self-monitoring of glucose levels, diet/exercise education, medication management, motivation for self-management, diabetes related problem solving, and lifestyle changes
Kazawa & Moriyama [48]	Self-management skills acquisition program on predialysis patients with diabetic nephropathy
Gagliardino et al. [49]	4-week structured education delivered by previously trained peers
Rygg et al. [50]	15-hour educational program, spread over three sessions, focusing on information about type 2 diabetes and its complications, diet, physical activity, and improving metabolic control
Yeung et al. [64]	2.5-year empowerment-based intervention involving 6 months low intensity and 24 months high-intensity education and support; the high-intensity education consisted of weekly group-based 75-minute support sessions
Davies et al. [63]	6-hour group education delivered in either one day or two half days equivalents
Pena-Purcell et al. [51]	2-hour 5 weekly sessions focusing on experiential and group activities to reinforce lesson concepts
Huang et al. [52]	Ongoing educational intervention with instructions on self-monitoring of glucose, medications, exercise, hygiene (foot care), and complication management
Song et al. [53]	6-week web-based intervention comprising an introduction, understanding diabetes, dietary management, exercise management, drug and test management, stress management, and foot care
Banister et al. [54]	4 hours of education followed by individual dietitian consults and monthly support meetings
Goudswaard et al. [55]	6-month 3-6 weekly sessions focusing on general information on diabetes, reinforcing compliance with actual medication, importance of physical exercise and losing body weight, and nutritional advice
Samuel-Hodge et al. [56]	12 months education: 8 months intensive phase consisting of 1 individual counselling visit, 12 group sessions, monthly phone contacts and 3 encouragement postcards, and 4 months reinforcement phase including telephone contacts
Glasgow et al. [57]	Internet-based educational program incorporating tailored self-management training and peer support
Rickheim et al. [58]	6 months education in 4 sequential sessions delivered at consistent time intervals
Deakin et al. [59]	The X-PERT program involving 6 weekly sessions, each lasting 2 hours long
Vincent [60]	8-week intervention consisting of 8-weekly 2-hour group sessions (including didactic content), cooking demonstrations, and group support sessions
Scain et al. [61]	8-hour structured group education program delivered in 4 sessions for 4 weeks, by a trained nurse educator
Two Feathers [62]	Racial and Ethnic Approaches to Community Health (REACH) Detroit partnership diabetes lifestyle intervention focusing on improving dietary, physical activity, and diabetes self-care behaviors

**Table 4:** Outcomes of DSME programs by type of care provider and setting of care.

Study	Setting of care	Care provider	Outcomes of SME
Merakou et al. [44]	Primary health clinic (diabetic outpatient clinic)	Primary physician	HbA1c (-), BMI (-)
Kazawa et al. [45]	Hospital	Specialist	HbA1c (=), BMI (=), self-efficacy (+)
Dyson et al. [46]	General practice surgeries	Primary care physician and practice nurse	HbA1C (-), BMI (-), physical activity (+)
Brunisholz et al. [47]	Primary care practice	General practitioner	HbA1c (-)
Kazawa and Moriyama [48]	Hospital and clinic	Primary physician	HbA1c (-), BMI (=), self- efficacy (+), diet (+), physical activity (+)
Gagliardino et al. [49]	Primary care institution	Primary physician	HbA1c (-), BMI (=)
Rygg et al. [50]	Primary care practice	Primary care provider	HbA1c (=), BMI (=)
Yeung et al. [64]	General medical practice	Primary physician	Health service utilization (=)
Davies et al. [63]	Primary care practices	Primary care provider	HbA1c (=), mental health (+), physical activity (=)
Pena-Purcell et al. [51]	Community health centre	Primary physician	HbA1c (-), self-efficacy (+)
Huang et al. [52]	Primary care clinic	Primary physician	HbA1c (-)
Song et al. [53]	Hospital	Specialist	HbA1c (-)
Banister et al. [54]	Community clinic	Clinic physician	HbA1c (-)
Goudswaard et al. [55]	General practice	Diabetes nurse and general practitioner	HbA1c (-)
Samuel-Hodge et al. [56]	Community health centre	Primary care clinician	HbA1c (-), diet (+), physical activity (=), mental health (+)
Glasgow et al. [57]	Primary care practices	Primary care physicians	Diet (+), physical activity (+), HbA1C (-) mental health (+)
Rickheim et al. [58]	General medical practice	General practitioner	HbA1c (-), physical activity (+), BMI (-), mental health (+)
Deakin et al. [59]	General medical practice	Primary care physician	HbA1c (-), BMI (-), physical activity (+)
Vincent [60]	Community health centre	Physician and nurse practitioner	Self-efficacy (=), physical activity (+), diet (=), HbA1C (=), BMI (-)
Scain et al. [61]	University hospital	Specialist	HBA1c (-), BMI (=)
Two Feathers [62]	Hospital and community health centre	Primary care physician and specialist	Diet (+), physical activity (=), HbA1c (-)

HbA1c: glycated hemoglobin; BMI: body mass index; (+): increase; (-): decrease; (=): no significant change.
